# Minimally Invasive Mitral Valve Repair in a Patient With Severe Scoliosis: Overcoming Anatomical Challenges

**DOI:** 10.7759/cureus.89774

**Published:** 2025-08-11

**Authors:** Laith Altawil, Mohammad Dayyeh, Bashar Abunnadi, Hani Qteishat, Abdul-Hakim Dayeh

**Affiliations:** 1 Department of Cardiothoracic Surgery, Specialty Hospital, Amman, JOR; 2 Department of Cardiothoracic Surgery, Jordan University of Science and Technology, Irbid, JOR

**Keywords:** minimal invasive cardiac surgery, mitral valve disease, mitral valve surgery, scoliosis, severe curve scoliosis, spinal deformities

## Abstract

Spinal deformities such as scoliosis present significant challenges in cardiac surgery due to thoracic anatomical distortion, restrictive lung physiology, and elevated perioperative risk. Minimally invasive mitral valve surgery offers reduced surgical trauma, shorter recovery, and lower complication rates compared to sternotomy, and may provide advantages in anatomically complex patients. However, such cases demand advanced technical precision, as distorted anatomy complicates cannulation, visualization, and valve exposure. We present the case of a 48-year-old patient with very severe thoracolumbar scoliosis (Cobb angle 130°) who underwent minimally invasive mitral valve repair via a 5-cm right mini-thoracotomy. Despite substantial anatomical challenges, the procedure was completed successfully using tailored cannulation strategies, heart repositioning techniques, and meticulous preoperative planning. The patient recovered without complications, was discharged on day four, and follow-up echocardiography confirmed normal valve function with marked symptomatic improvement. This case highlights the feasibility of minimally invasive mitral valve repair in anatomically extreme patients when performed with individualized planning, intraoperative adaptability, and institutional expertise.

## Introduction

Scoliosis, defined by the Scoliosis Research Society as a lateral spinal curvature exceeding 10° measured by the Cobb method on standing radiographs, is primarily a musculoskeletal disorder but can have profound cardiopulmonary consequences when severe or thoracic in location [1,2]. Significant thoracic distortion can compress cardiac structures, reduce lung volumes, and produce restrictive respiratory physiology, factors that complicate perioperative assessment and surgical planning [1-3].

Our patient presented with severe mitral regurgitation requiring surgical intervention, but was declined at multiple centers due to the risks posed by her extreme spinal deformity. She sought a minimally invasive approach at our institution, as minimally invasive mitral valve surgery offers advantages over conventional sternotomy, including reduced trauma, shorter recovery, and lower complication rates [4]. However, in patients with severe scoliosis, distorted thoracic anatomy and displaced cardiovascular structures create major challenges for such procedures, narrowing operative corridors and complicating visualization, cannulation, and exposure. These cases require not only institutional expertise but also advanced preoperative imaging and individualized surgical strategies to ensure safe and effective outcomes.

## Case presentation

A 48-year-old patient presented with New York Heart Association (NYHA) Class IV symptoms, including progressively worsening dyspnea, fatigue, and palpitations with minimal exertion. Transthoracic and transesophageal echocardiography confirmed severe mitral regurgitation due to flail of the posterior leaflet secondary to ruptured chordae tendineae. Left ventricular systolic function was preserved, with an ejection fraction of 55%, and there was marked left atrial enlargement. Surgical intervention was indicated.

**Figure 1:**
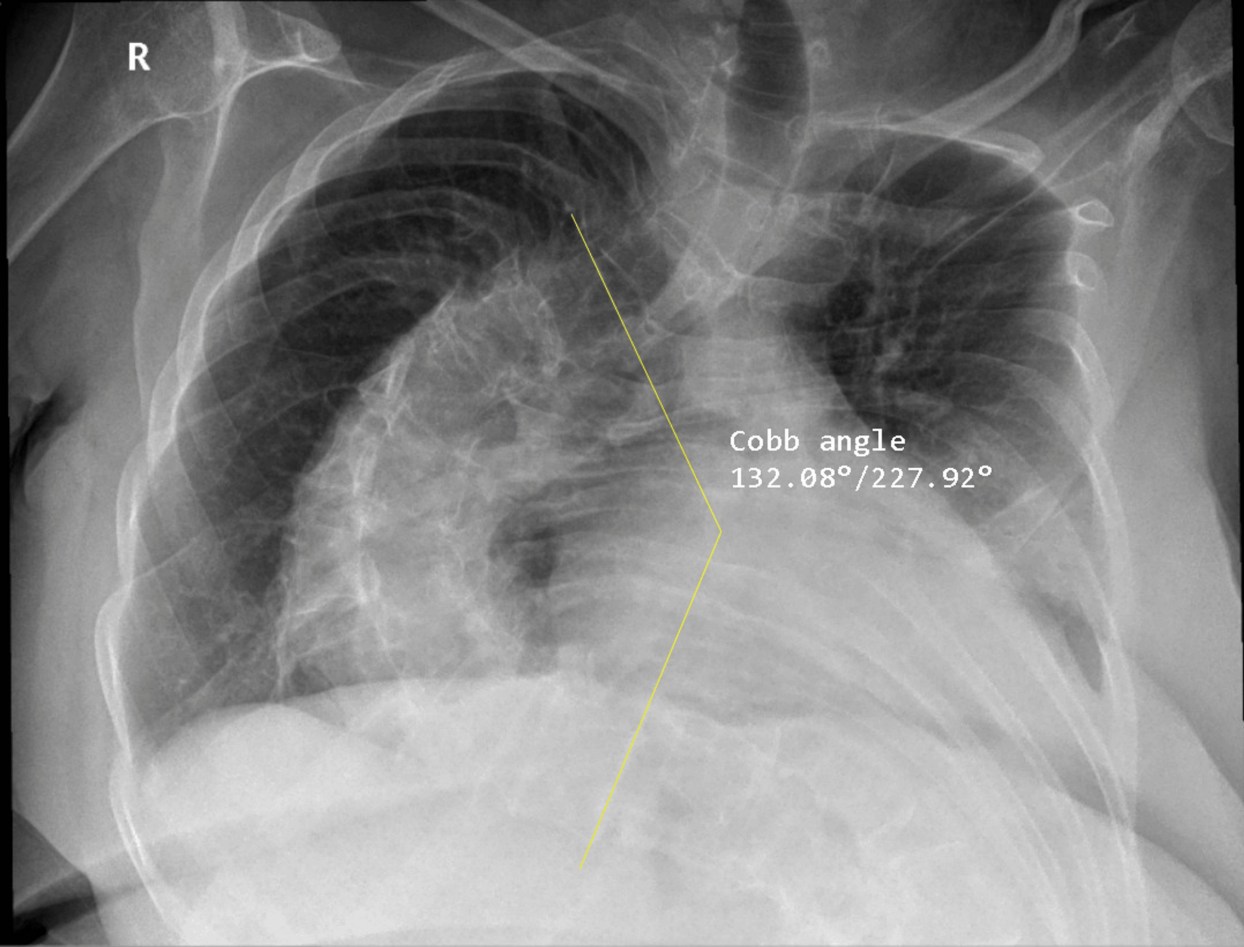
Chest radiograph. Anteroposterior view demonstrating a marked S‑shaped thoracolumbar scoliosis with right‑sided thoracic convexity. The Cobb angle measures 130°, consistent with very severe scoliosis.

The patient’s comorbidities were primarily the result of long-standing poliomyelitis, which had led to severe thoracolumbar scoliosis with a Cobb angle of 130° (Figure 1), restrictive lung disease (FEV₁/FVC ratio of 74% with reduced total lung capacity and diffusing capacity for carbon monoxide), and marked spinal deformity necessitating assistance for ambulation with heavy reliance on the upper limbs. Despite the pronounced deformity and the presence of ruptured tendinous chords, there were no stigmata of Marfan syndrome or any other connective tissue disorder. The extent of the spinal deformity rendered postoperative recovery after sternotomy particularly limiting, and the patient had been declined for surgery at multiple cardiac centers owing to the perceived prohibitive operative risk.

Preoperative imaging revealed profound spinal curvature, distortion of the thoracic cavity, and significant displacement of the heart and great vessels, all anticipated to complicate cannulation and valve exposure. The thoracoabdominal aorta and vena cava were tortuous, the ascending aorta was displaced to the left, and the elevated thoracic spine obstructed direct visualization of the left atrium. A detailed preoperative plan was developed, including the use of specialized wires and catheters to facilitate cannulation and strategies to optimize exposure despite the distorted anatomy.

**Figure 2:**
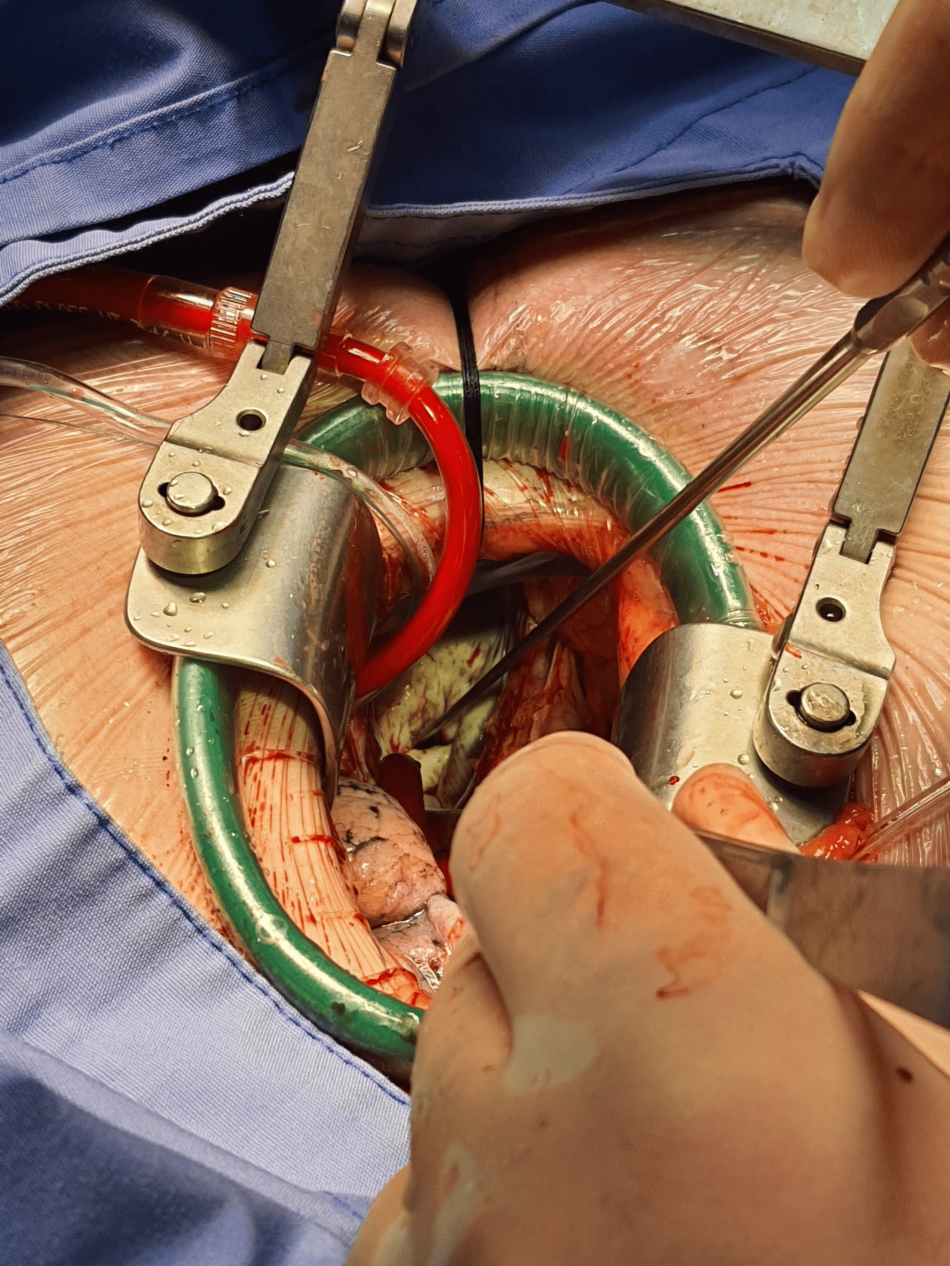
Minimally invasive thoracotomy incision Right anterolateral 5‑cm mini‑thoracotomy performed through the fourth intercostal space for mitral valve repair. The white structure visible in the surgical field is the mitral valve. A left ventricular vent is also in place. Arterial and venous cannulation were performed via the femoral route.

The patient underwent minimally invasive mitral valve repair (MIMVR) under general anesthesia via a 5‑cm right anterolateral mini‑thoracotomy through the fourth intercostal space with femoral arterial and venous cannulation (Figure 2). Venous cannulation was initially unsuccessful with a standard guidewire due to resistance in the superior vena cava. A 0.035‑inch hydrophilic guidewire (Terumo®, Tokyo, Japan) was then used to traverse the tortuous path, later exchanged for a Teflon‑coated stiff guidewire (Cook Medical®, USA) to advance a multistage venous cannula (Medtronic Biomedicus®, Medtronic Inc., Minneapolis, MN, USA). Arterial cannulation was achieved uneventfully using a standard Teflon guidewire and arterial cannula (Medtronic Biomedicus®, Medtronic Inc., Minneapolis, MN, USA).

After thoracotomy, the thoracic spine lay directly between the heart and the surgical field, requiring a stay suture to shift the heart anteriorly and toward the right, enabling placement of the cross-clamp and cardioplegia needle. Once adequate exposure was achieved, the repair proceeded routinely. The mitral valve was reconstructed using two artificial chordae tendineae anchored to the papillary muscle and the P2 segment of the posterior leaflet, followed by placement of annuloplasty sutures and implantation of an Edwards Cosgrove® annuloplasty band (Edwards Lifesciences, Irvine, CA, USA).

The procedure was completed without complications. The patient ambulated on postoperative day one and was discharged on day four with standard care instructions. Early outpatient follow-up showed marked symptomatic and functional improvement. Transthoracic echocardiography confirmed normal function of the repaired valve, with no paravalvular leak, residual regurgitation, or structural abnormalities. Recovery remained uncomplicated, with no readmissions or further interventions required.

## Discussion

This case demonstrates how extreme thoracolumbar scoliosis (Cobb angle 130°) can profoundly alter cardiothoracic anatomy, producing severe thoracoabdominal distortion, vessel tortuosity, and leftward displacement of the ascending aorta, with the rotated thoracic spine obstructing direct visualization of the left atrium. Such challenges demand meticulous preoperative imaging to define anatomical relationships and guide operative planning, as well as tailored intraoperative adaptations. In this instance, femoral venous cannulation was facilitated by hydrophilic and stiff Teflon-coated guidewires, while stay sutures were used to reposition the heart anteriorly and laterally, enabling optimal exposure of the mitral valve.

This case represented a complex decision-making process, as the patient had been declined for surgery at multiple institutions due to the perceived operative risk. Management options included mitral valve repair via conventional sternotomy, a minimally invasive approach, or transcatheter mitral clip repair. A multidisciplinary team discussion involving cardiac surgery and cardiology carefully evaluated these options, taking into account patient-specific factors, procedural risks and benefits, and long-term outcomes. Transcatheter mitral valve repair, although less invasive, is generally considered palliative rather than curative, with durability and survival benefits that are less favorable in younger patients [4]. In this 48-year-old patient with preserved left ventricular function, the procedure fell outside the official indications for mitral clip repair, and surgical repair was deemed to offer greater durability and long-term survival. Between the surgical approaches, the patient’s dependence on upper limbs for mobility made sternal recovery particularly limiting, and her restrictive lung disease increased the risk of postoperative respiratory complications with sternotomy. The choice of a minimally invasive approach was further supported by our institutional expertise in complex mitral valve repair and the patient’s informed preference after fully understanding the risks, benefits, and alternative treatment options. Current European Society of Cardiology and American Heart Association/American College of Cardiology guidelines endorse mitral valve repair for regurgitation but do not provide specific recommendations for patients with severe thoracic deformity, leaving operative strategy to the judgement of experienced surgical teams [5,6].

This case contributes to a growing body of evidence, including reports by Bartolozzi et al. and Kitamura et al., demonstrating that severe spinal deformities do not preclude successful MIMVR when meticulous preoperative planning, imaging, and flexible intraoperative techniques are applied [7,8]. It reinforces that with multidisciplinary collaboration and specialized expertise, MIMVR can be performed and reproduced in patients traditionally viewed as high risk for conventional surgery. Nonetheless, the rarity of such cases and the absence of formal guidelines highlight the need for larger series and registries to refine operative strategies and inform future recommendations for this complex population.

## Conclusions

This case underscores the value of tailoring surgical strategies to the individual, prioritising durability and long-term outcomes over palliative alternatives in suitable patients. The result reflects the impact of institutional expertise, careful risk-benefit evaluation, and meaningful patient involvement in decision-making. Beyond technical success, it illustrates how expanding the application of minimally invasive approaches can offer selected high-risk patients the chance of restored function and sustained quality of life.
